# Epidemiology of Diabetic Ketoacidosis in the Waikato Region of New Zealand: 2000-2019

**DOI:** 10.1155/2023/4715783

**Published:** 2023-09-09

**Authors:** Lynne Chepulis, Valentina Papa, Chunhuan Lao, Justina Wu, Cinthia Minatel Riguetto, Joanna M. McClintock, Ryan G. Paul

**Affiliations:** ^1^Medical Research Centre, Te Huataki Waiora School of Health, University of Waikato, Hamilton, New Zealand; ^2^Faculty of Medical and Health Sciences, University of Auckland, New Zealand; ^3^Waikato Regional Diabetes Service, Te Whatu Ora Health New Zealand, Hamilton, New Zealand

## Abstract

**Aims:**

Diabetic ketoacidosis (DKA) is not well characterised in New Zealand. This study is aimed at characterising the change in epidemiology and severity of DKA from 2000 to 2019 at a tertiary hospital in the Waikato region of New Zealand.

**Methods:**

A retrospective clinical data review of all patients admitted to Waikato District Health Board hospitals with DKA was undertaken. Characteristics and severity of DKA were assessed by type of DKA admission (diagnosed at admission, nonrecurrent, and recurrent), ethnicity, social deprivation, intensive care unit (ICU) admission, and length of hospital stay, with linear regression reporting on changes over time.

**Results:**

There were 1254 admissions for DKA (564 individual patients), two-thirds being recurrent events. Nonrecurrent DKA patients were younger, whilst recurrent admissions for DKA were associated with T1D, female gender, greater socioeconomic deprivation, and rural living (all *P* values < 0.01). DKA admission increased 8-fold between 2000 and 2019, mostly due to an increased number of recurrent events, particularly in Māori and female patients (*P* < 0.001). ICU admissions increased over time (*P* < 0.001) whilst length of hospital stay trended down (*P* = 0.031).

**Conclusions:**

The rise in recurrent DKA is concerning, particularly in youth and indigenous Māori. Healthcare inequities need to be addressed, including adequate access to mental health support to ensure optimal outcomes for all patients with diabetes.

## 1. Introduction

Diabetic ketoacidosis (DKA) is an acute and potentially fatal metabolic complication of type 1 diabetes (T1D) and type 2 diabetes (T2D) with severe insulin deficiency and pancreatogenic diabetes [1, 2]. DKA may be the first presentation of previously unknown diabetes mellitus or may be a nonrecurrent or recurrent event in those with established diabetes mellitus due to either omission of insulin and/or intercurrent stressors [3]. Although DKA is largely preventable, it is the leading cause of mortality in children with T1D and is associated with long-term sequelae, including cognitive and renal impairment in both children and adults [4–6]. Recurrent presentations of DKA are of particular concern. Risk factors for recurrent DKA include T1D, younger age, low socioeconomic status, suboptimal glycaemic control, alcohol and recreational drug use, insulin pump use, disordered eating, and other mental health disorders [7–9].

Whilst the incidence of DKA as the first presentation of diabetes appears to have remained stable for many years in New Zealand and worldwide, the incidence of DKA in those with established diabetes appears to have significantly increased over the past 20 years [1, 2, 4, 10]. This increase is primarily due to recurrent DKA [1, 10], but there is a significant variation in the incidence of DKA between studies, with many reporting no change in incidence over time [11–16]. These differences appear to be mainly due to differences in the demographics of the populations [14–16], different definitions of DKA [17], lack of differentiation between recurrent DKA and nonrecurrent DKA at diagnosis, and differences in health systems [18, 19].

The Waikato region in New Zealand is a predominantly urban area that comprises approximately 400,000 people, of whom 21% identify as Māori, the Indigenous people of New Zealand [20]. The prevalence of T1D and T2D in the Waikato population is approximately 0.3% [20] and 6.3% [21], respectively. In the Waikato region, as seen internationally, Indigenous Māori and those more socioeconomically deprived with either T1D or T2D have a higher glycated haemoglobin (HbA1c) [20, 21], putting them at greater risk of a DKA event. The demographic breakdown of DKA in a New Zealand paediatric population and the outcomes of DKA in a New Zealand adult population have been well described [13, 16, 22], but the epidemiology of DKA at diagnosis and recurrent DKA across the lifespan in New Zealand has not. Therefore, the aim of this study was to characterise the epidemiology of DKA in the Waikato region and to identify any disparities in the incidence and severity of DKA.

## 2. Materials and Methods

This study was a retrospective clinical data review of all patients who were admitted to Te Whatu Ora Waikato hospitals (previously Waikato District Health Board) with DKA between 1st January 2000 and 31st December 2019. DKA was defined as pH < 7.3, serum bicarbonate (HCO_3_^−^) < 18 mmol/L, ketonaemia (blood beta‐hydroxybutyrate ≥ 0.6 mmol/L), and hyperglycaemia [17]. The following data were collated from the clinical records: date of DKA onset and related hospital admission, age at admission, ethnicity, venous pH and HCO_3_^−^ levels, admission to the intensive care unit (ICU) (yes/no), length of hospital stay, mortality during admission, and mortality within 12 months. In addition, patient socioeconomic status was determined using the patient's address at the time of admission, and rural/urban locality status was categorised according to Statistics New Zealand, New Zealand's official data agency [23]. Approval for this study was given by the Waikato District Health Board Clinical Audit Support Unit (ref #4161).

### 2.1. Data Analysis

The types of DKA at admission were categorised into three groups: DKA with diagnosis of diabetes at admission, nonrecurrent DKA (known diabetes with one episode of DKA in the study period), and recurrent DKA (two or more admissions within the study period). The patient characteristics of each group were then reported by gender, ethnicity (Māori vs. non-Māori), age group (≤5, 5-14, 15-24, 25-34, 35-44, and ≥45 years), rurality (rural vs. urban), New Zealand deprivation quintile (1-5, where quintile 5 represents the highest deprivation), type of diabetes (T1D, T2D, and unknown), venous pH levels, HCO_3_^−^ levels, and length of hospital stay (LOS). For continuous variables, data ware presented as the median and interquartile range (IQR). Ethnicity was grouped as Māori and non-Māori with prioritisation for Māori with multiple ethnicities, and the differences between groups were compared by chi-square test for categorical variables and independent sample *t*-test for continuous variables. Significance was accepted at a level of *P* value < 0.05 (*P* < 0.05).

The total number of DKA admissions and the numbers by type of admission were reported over time. Linear regression analysis was used to examine the effect of time on the number of DKA admissions and to identify the contributions from different types of DKA admissions. Logistic regression analysis was used to estimate the odds ratios and 95% confidence interval (CI) of being admitted to ICU, having recurrent DKA, and dying within 12 months for Māori and non-Māori patients (all as markers of DKA severity). Trends are reported for LOS and ICU admissions over time (DKA at diagnosis, nonrecurrent, and recurrent DKA). The analyses were carried out before and after adjustment for age, gender, NZ deprivation quintile, rurality, year of admission, and types of admissions (not for the regression for having recurrent DKA). For the regression of being admitted to ICU and dying within 12 months, the analysis was performed at hospital admission level. All DKA admissions were included. For the regression of having recurrent DKA, the analysis was performed at patient level. Only the first record for each patient was included, and if the patients had more than one admission, they were classified as having recurrent DKA.

## 3. Results

During the study period, there were 1254 admissions for DKA in 564 individual patients (Tables 1 and 2). Of these, 156 (12.4%) were diagnosed with diabetes at presentation with DKA, 247 (19.7%) had known diabetes with one episode of DKA in the study period (nonrecurrent DKA), and 851 (67.9%) were recurrent DKA events. Of all DKA admissions, one quarter (26.2%) occurred in Māori patients and 73.8% in non-Māori patients, and the incidence of events was relatively comparable for men (48.3%) and women (51.7%; *P* = 0.23). The median age at admission was 24 years (IQR: 17–38 years). Patients who presented with DKA as their first presentation of diabetes were younger (48.1% were aged <15 years; *P* < 0.001), whilst those with recurrent DKA events were more likely to be aged 15–24 years (*P* < 0.001). Socioeconomic deprivation also influenced the likelihood of a DKA event, with over half of the DKA cases occurring in patients from NZ deprivation quintiles 4 and 5 (Tables 1 and 2). This was consistent regardless of whether this was the first DKA presentation with diabetes or a recurrent DKA event. Not surprisingly, more than 90% of DKA events occurred in those with T1D. Of the 564 patients, approximately two-thirds had only one admission for DKA (*n* = 370; 65.6%; Table 2) during the study period. A further 116 patients (20.6%) had two or three DKA admissions, and 78 (13.8%) had four or more admissions. Those with four or more DKA admissions were more likely to be 15-24 years of age (Table 2). Overall, a greater number of admissions for DKA were associated with T1D, female gender, greater socioeconomic deprivation, and rural living (all *P* values < 0.01). Tables 1 and 2 summarize the characteristics of DKA events in each subgroup and the patient characteristics by number of DKA admissions, respectively.

Almost one in five patients with recurrent DKA was admitted to ICU (*n* = 105; 18.6%). Five patients (0.9%) died during their admission (one Māori (recurrent DKA) and 4 non-Māori (two recurrent and two nonrecurrent events)), and a further 35 patients died within the following 12 months (1-year mortality, 7.1%). There was no significant difference between Māori and non-Māori patients being admitted to ICU or dying within 12 months before or after adjustment for age, gender, NZ deprivation quintile, rurality, year of admission, and type of admissions (Table 3).

**Figure 1:**
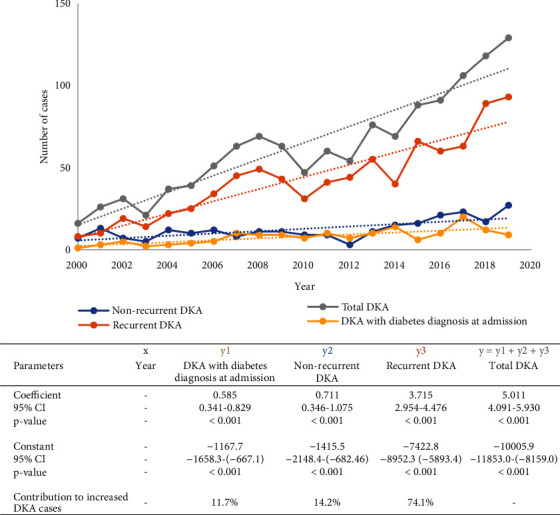
Number of DKA admissions over time and linear regression results. DKA = diabetic ketoacidosis; CI = confidence interval.

The number of DKA admissions increased 8-fold over time (Figure 1), from 16 cases in 2000 to 129 cases in 2019. The linear regression showed that three quarters of this increase were due to increase in recurrent DKA events, though DKA with diabetes admission and nonrecurrent DKA rates also increased significantly over time (Figure 1). Māori patients were more likely to have recurrent DKA than non-Māori patients, with an unadjusted odds ratio of 1.93 (1.30–2.88) and an adjusted odds ratio of 1.95 (1.27–3.00). Women were also more likely than men to have recurrent DKA events with an odds ratio of 1.61 (1.13–2.28; Table 3). Rural living was not associated with an increased risk of recurrent DKA but was associated with a greater risk of admission to ICU (*P* < 0.05).

**Figure 2:**
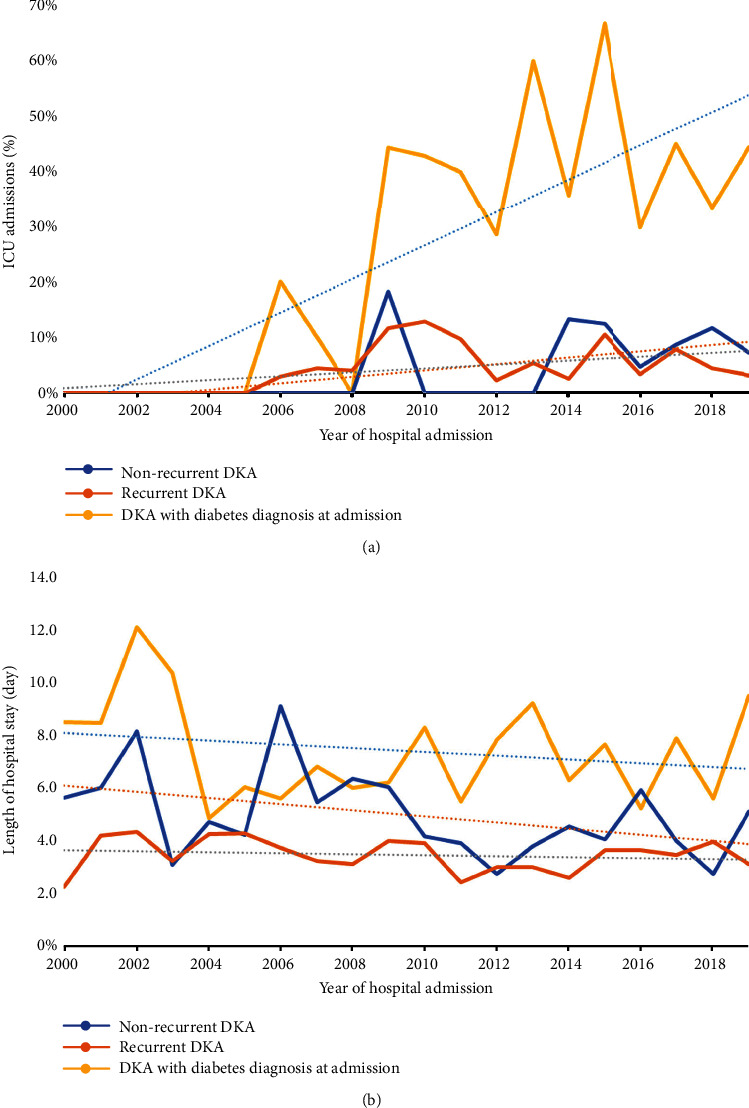
Proportion of intensive care unit (ICU) admissions (a) and mean length of hospital stay (LOS) (b) due to different types of diabetic ketoacidosis (DKA) presentation.

Overall, there was no change in median venous pH or mortality over time, though the proportion of patients admitted to ICU trended up between 2000 and 2019 (*P* < 0.001; OR 1.14 (1.05–1.23)). This was primarily due to an increased proportion of ICU admissions in those diagnosed with DKA at the time of hospital admission (Figure 2(a)). In contrast, mean LOS trended mildly downward over time (Figure 2(b); *P* = 0.031), Overall, severity of disease did not differ by ethnicity or the level of socioeconomic deprivation.

## 4. Discussion

Our study shows that there has been a dramatic increase in number of presentations of DKA in the Waikato region of New Zealand over time and this has occurred despite the overall number of patients with diabetes (type 1 and type 2 combined) in the region only rising by approximately 6% during this same time period [24]. Importantly, this rise appears to not be due to changes in overall glycaemic control [25] but instead seems to be related to an increase in recurrent DKA events in young adults and/or in those living in areas of higher deprivation. Such findings agree with that presented elsewhere that recurrent DKA associates with emerging adulthood [5, 7, 22], and this is mainly due to a higher overall HbA1c, reduced access to healthcare, and adherence to insulin treatment [7, 22, 26]. Unfortunately, psychosocial screening is not routine for admissions to hospital in New Zealand, though this could provide further insight into these DKA admissions, given the higher rates of low mood and disordered eating in young adults and adolescents with diabetes (particularly T1D) [27]. Through the identification of mental health concerns and diabetes-related distress, appropriate interventions could be introduced, potentially reducing the “recurrent” nature of DKA. Socioeconomic deprivation has also been shown to strongly correlate with a higher incidence of DKA in other large studies [28]. In addition to this, lower health literacy [28] and reduced access to high-quality diabetes care, healthy foods, and diabetes technology (e.g., insulin pumps and continuous glucose monitoring) [29, 30] can all contribute to an increased likelihood of glycaemic dysfunction (and therefore increased DKA risk). The contribution of these factors needs to be urgently evaluated. Further, addressing these inequities needs to be a priority for healthcare in New Zealand if we are to see reductions in the rate of DKA presentations, and it will be interesting to observe the future rates of change for DKA presentations from these population groups following national restructuring of New Zealand health system in 2022.

Importantly, despite the exponential increase seen in recurrent DKA presentations, there has not been any increase in several parameters of DKA severity. Indeed, the mortality rates observed in our study are similar to other national and international studies [4, 31, 32], and LOS appears to have decreased over time, as it has at another tertiary hospital in New Zealand [22]. These findings may be indicative of improvement in initial clinical care but are also representative of a similar mode of healthcare provision between these two centres which may differ from that seen in other countries [11]. However, ICU admissions were either stable or increasing in our cohort which agrees with that report elsewhere [31]. We suggest that in our study, this was likely due to the reduced ability of medical wards to manage mild DKA.

Our data also indicates that the rate of presentation and severity of DKA does not appear to be worse for Māori patients. It has been suggested that this may be due to the fact that most DKA admissions in our study associated with T1D and Māori are only half as likely as non-Māori to be affected by T1D [33]. However, it is encouraging to see that there does not appear to be any inequity in treatment after presentation to hospitals in this region.

The main strength of our study is that we were able to report on 20 years of continuous data from a large, regional hospital with good representation of indigenous Māori, though we do note study limitations. Firstly, our findings may not be representative of all New Zealand because of the different age, ethnic, and rural demographics throughout the country. Secondly, whilst we are able to discuss our data in the context of national data that reports yearly on the number of diabetes patients within our region [24], we are unable to report on how the demographic and obesity rates may have changed between 2000 and 2019 (with regard to age, sex, and ethnicity) and how this may have influenced the results seen. Third, we note that our study lacked both HbA1c data and T1D prevalence data. This makes it difficult to make comparisons to other literature reporting on DKA rates. These factors, collectively, should all be explored in later studies through the use of population/census and clinical/laboratory information. Further, we note the possibility that some patients coded as “nonrecurrent DKA” may have had an additional DKA event outside of the study period and that DKA severity could have been more precisely defined [34]. In particular, the inclusion of admission glucose values would have yielded useful information on the epidemiology of euglycaemic DKA, and this should be explored in further studies.

However, our data do show that there is clearly a very strong need to reduce the risk factors for recurrent DKA in our local population, particularly in young adults and those from lower socioeconomic backgrounds. Based on our findings and those of others, we suggest that it would be useful to screen all patients who are admitted with recurrent DKA for mental health concerns (including substance abuse) and diabetes-related distress. In addition, these patients should receive appropriate input from multidisciplinary team members as soon as possible, including from psychology, dietitian, social, youth and cultural workers, and specialist diabetes services in either the inpatient or outpatient setting.

## Figures and Tables

**Table 1:** DKA event characteristics by DKA subgroup.

Factors	1: DKA with diabetes diagnosis at admission		2: nonrecurrent DKA		3: recurrent DKA		Total DKA		*P* value			
									3 groups	1 vs. 2	1 vs. 3	2 vs. 3
Gender												
Female	66	42.3%	121	49.0%	461	54.2%	648	51.7%	0.016	0.190	0.006	0.151
Male	90	57.7%	126	51.0%	390	45.8%	606	48.3%				
Ethnicity												
Māori	34	21.8%	48	19.4%	246	28.9%	328	26.2%	0.005	0.566	0.068	0.003
Non-Māori	122	78.2%	199	80.6%	605	71.1%	926	73.8%				
Age group												
<5 years	16	10.3%	0		1	0.1%	17	1.4%	<0.001	<0.001	<0.001	<0.001
5-14 years	59	37.8%	21	8.5%	81	9.5%	161	12.8%				
15-24 years	34	21.8%	62	25.1%	363	42.7%	459	36.6%				
25-34 years	13	8.3%	30	12.1%	192	22.6%	235	18.7%				
35-44 years	14	9.0%	30	12.1%	121	14.2%	165	13.2%				
≥45 years	20	12.8%	104	42.1%	93	10.9%	217	17.3%				
Rurality												
Rural	36	23.1%	60	24.3%	192	22.6%	288	23.0%	0.850	0.780	0.888	0.569
Urban	120	76.9%	187	75.7%	659	77.4%	966	77.0%				
NZ deprivation (quintile)^1^												
1	20	12.8%	18	7.3%	71	8.3%	109	8.7%	0.327	0.365	0.301	0.389
2	13	8.3%	20	8.1%	96	11.3%	129	10.3%				
3	27	17.3%	38	15.4%	130	15.3%	195	15.6%				
4	36	23.1%	68	27.5%	192	22.6%	296	23.6%				
5	60	38.5%	103	41.7%	362	42.5%	525	41.9%				
Type of diabetes mellitus^2^												
Type 1	140	93.3%	184	74.8%	819	96.4%	1143	91.7%	<0.001	<0.001	<0.001	<0.001
Type 2	10	6.7%	62	25.2%	31	3.6%	103	8.3%				
pH (median+IQR)	7.20	(7.12-7.28)	7.20	(7.08-7.27)	7.20	(7.08-7.27)	7.20	(7.08-7.27)	0.480	0.654	0.412	0.723
HCO_3_^−^ (median+IQR)	12.0	(8.78-16.73)	12.0	(8.00-16.00)	12.0	(8.20-16.20)	12.0	(8.30-16.20)	0.860	0.954	0.881	0.920
LOS (days) (median+IQR)	6.70	(4.66-8.24)	3.80	(1.90-6.84)	2.70	(1.77-4.16)	3.00	(1.87-5.47)	<0.001	<0.001	<0.001	<0.001
Total	156	12.4%	247	19.7%	851	67.9%	1254	100%				

^1^Quintile 1 represents the least deprivation, and quintile 5 represents the highest deprivation. ^2^Six cases of unknown diabetes type. DKA = diabetic ketoacidosis; HCO_3_^−^ = bicarbonate; IQR = interquartile range; LOS = length of stay.

**Table 2:** Patient characteristics by number of diabetic ketoacidosis (DKA) admissions.

Factors	Total	Only 1 DKA		2-3 DKAs		4+ DKAs		*P* value
Gender								
Female	282	170	60.3%	72	25.5%	40	14.2%	0.01
Male	282	200	70.9%	44	15.6%	38	13.5%	
Ethnicity								
Māori	132	71	53.8%	41	31.1%	20	15.2%	0.001
Non-Māori	432	299	69.2%	75	17.4%	58	13.4%	
Age group (at first admission)								
<5 years	16	15	12.3%	1	0.8%	0	0.0%	<0.001
5-14 years	106	62	50.8%	30	24.6%	14	11.5%	
15-24 years	156	86	55.1%	30	19.2%	40	25.6%	
25-34 years	76	42	55.3%	22	28.9%	12	15.8%	
35-44 years	62	42	67.7%	13	21.0%	7	11.3%	
≥45 years	148	123	83.1%	20	13.5%	5	3.4%	
Rurality								
Rural	164	88	53.7%	45	27.4%	31	18.9%	<0.001
Urban	400	282	70.5%	71	17.8%	47	11.8%	
NZ deprivation (quintile)^1^								
1	48	34	70.8%	5	10.4%	9	18.8%	<0.001
2	54	31	57.4%	13	24.1%	10	18.5%	
3	88	60	68.2%	20	22.7%	8	9.1%	
4	146	98	67.1%	31	21.2%	17	11.6%	
5	228	147	64.5%	47	20.6%	34	14.9%	
Type of diabetes mellitus								
Type 1	474	292	61.6%	106	22.4%	76	16.0%	<0.001
Type 2	83	72	86.7%	9	10.8%	2	2.4%	
Unknown	7	6	85.7%	1	14.3%			
Total	564	370	65.6%	116	20.6%	78	13.8%	

^1^Quintile 1 represents the least deprivation, and quintile 5 represents the highest deprivation.

**Table 3:** Odds ratio of ICU admission, recurrent DKA, and mortality within 12 months.

Factors	Being admitted to ICU	Having recurrent DKA	Dying within 12 months
Age at admission (continuous)	0.96 (0.94-0.98)^∗∗∗^	0.98 (0.97-0.99)^∗∗∗^	1.08 (1.06-1.10)^∗∗∗^
Gender			
Female	Reference	Reference	Reference
Male	0.43 (0.23-0.79)^∗∗^	0.68 (0.47-0.98)^∗^	0.90 (0.40-2.02)
NZ deprivation quintile^1^			
1	Reference	Reference	Reference
2	0.37 (0.09-1.54)	1.76 (0.75-4.13)	1.23 (0.25-6.11)
3	0.77 (0.28-2.13)	1.07 (0.48-2.38)	0.45 (0.08-2.46)
4	0.63 (0.22-1.75)	1.17 (0.55-2.46)	0.56 (0.14-2.20)
5	0.69 (0.26-1.80)	1.27 (0.62-2.62)	0.52 (0.14-1.92)
Rurality			
Urban	Reference	Reference	Reference
Rural	2.16 (1.17-3.99)^∗^	1.38 (0.89-2.13)	0.52 (0.18-1.47)
Ethnicity			
Non-Māori	Reference	Reference	Reference
Māori	1.40 (0.74-2.65)	1.95 (1.27-3.00)^∗∗^	1.56 (0.63-3.86)
Year (continuous)	1.06 (1.00-1.12)^∗^	0.94 (0.91-0.97)^∗∗∗^	1.10 (1.01-1.19)
Type of admission			
Non-recurrent	Reference	—	Reference
Recurrent	0.66 (0.34-1.28)	—	0.52 (0.22-1.21)

^1^Quintile 1 represents the least deprivation, and quintile 5 represents the highest deprivation. ^∗^ < 0.05, ^∗∗^ < 0.01, and ^∗∗∗^ < 0.001. DKA = diabetic ketoacidosis; ICU = intensive care unit.

## Data Availability

The data used to support the findings of this study are restricted by Te Whatu Ora Health New Zealand in order to protect patient privacy. Data are available from Dr. Ryan Paul for researchers who meet the criteria for access to confidential data.
